# P396: Balancing ventilation and energy use in hospitals: a case study of bioaerosol transport in healthcare environments

**DOI:** 10.1186/2047-2994-2-S1-P396

**Published:** 2013-06-20

**Authors:** K Grosskopf

**Affiliations:** 1 University of Nebraska, Lincoln, Nebraska, USA

## Introduction

Hospitals are among the most energy intensive buildings in industrialized countries, using more than two-thirds of total energy consumption to maintain climate control and indoor air quality (IAQ). Yet, hospital acquired infections (HAIs) afflict approximately 8% of the in-patient population and claim more than 115,000 lives each year in Europe and the U.S. alone.

## Methods

A series of 4 tests were conducted in an actual hospital to observe removal rates and containment of synthetic bioaerosols with respect to air change rate, air pressure differential and door position in a general patient room, and, an isolation patient room.

## Results

Air change rates were not found to be effective in proportionately reducing aerosol concentrations within patient rooms when the aerosol was *continuously* released. Specifically, increasing mechanical ventilation rates from 2.5 to 5.5 air changes per hour (ACH) reduced aerosol concentrations only 30% on average. An air pressure differential of 2.5Pa, however, was found to be effective in containing aerosol transport from patient rooms to adjacent corridors *except* in cases where isolation anterooms were negatively pressurized with respect to isolation spaces. Door position and door motion was also found to have a significant effect on aerosol containment.

## Disclosure of interest

None declared

